# MODY PDX1^P33T^: a mouse model reveals phenotypic divergence from human disease

**DOI:** 10.3389/fendo.2025.1680893

**Published:** 2025-10-22

**Authors:** Aliona Harten, Maximilian R. Schmidtke, Florian Giesert, David A. Skerrett-Byrne, Raffaele Teperino, Gerhard K. H. Przemeck, Martin Hrabě de Angelis

**Affiliations:** ^1^ Institute of Experimental Genetics, Helmholtz Zentrum München, German Research Center for Environmental Health, Neuherberg, Germany; ^2^ German Center for Diabetes Research (DZD), Neuherberg, Germany; ^3^ Institute of Stem Cell Research, Helmholtz Zentrum München, German Research Center for Environmental Health, Neuherberg, Germany; ^4^ Infertility and Reproduction Research Program, Hunter Medical Research Institute, New Lambton Heights, NSW, Australia; ^5^ School of Biomedical Sciences and Pharmacy, College of Health, Medicine and Wellbeing, University of Newcastle, Awabakal Country, Callaghan, NSW, Australia; ^6^ Chair of Experimental Genetics, TUM School of Life Sciences, Technische Universität München, Freising, Germany

**Keywords:** PDX1, MODY, MODY4, diabetes mellitus, mouse model, point mutation, phenotypic divergence

## Abstract

**Introduction:**

Maturity-onset Diabetes of the Young (MODY) is a rare form of diabetes and arises from mutations in key regulatory genes of the pancreatic beta cell, leading to their functional impairment and early-onset diabetes. Research into PDX1-MODY, a form of MODY caused by mutations in the *PDX1* gene, enhances understanding of gene-specific mechanisms underlying glucose dysregulation and provides insights into possible approaches to restore normal metabolic function. However, no currently published mouse model accurately depicts the genetic cause of PDX1-MODY in human patients.

**Methods:**

Using CRISPR-Cas9 technology, we generated the first mouse model carrying one of the most prevalent pathological PDX1 point mutation found in human patients, P33T, and conducted an 18-week *in vivo* phenotyping experiment assessing homozygous PDX1^P33T^ and wild-type littermates on both chow and high fat diet (HFD). Additionally, transcriptomic and proteomic analyses were performed on isolated pancreatic islets. Islet architecture was investigated via fluorescent microscopy.

**Result:**

Contrary to expectations, our comprehensive phenotypic analysis of the mouse model carrying the homozygous PDX1^P33T^ mutation revealed no significant differences in metabolic parameters compared to wild-type controls, and no pathological outcomes were observed as seen in human patients. Notably, male PDX1^P33T^ mice exhibited an increase in islet size and number on chow diet, with omics analyses suggesting reprogramming toward stress resilience, but failed to adapt respectively on HFD.

**Discussion:**

Our work indicates substantial differences between mouse and human PDX1 function in the pancreas. Further refinement of animal models is necessary to better elucidate the pathophysiology of PDX1-MODY.

## 1 Introduction

The transcription factor Pancreatic and Duodenum Homeobox 1 (PDX1) is a master regulator of the pancreas in both embryonic development and adulthood. During embryogenesis, it directs the commitment of progenitor cells to become endocrine cells and subsequently beta cells, maintaining them in their differentiated state during adult stages (1). PDX1 regulates the expression of numerous genes relevant for proper beta cell function, including *Ins1*, *Ins2*, *Mafa*, and *Pdx1* itself. It contains a DNA-binding domain and a transactivation domain, which facilitates interactions with cofactors, including the formation of heterodimers (2).

In humans, heterozygous *PDX1* mutations are known to cause PDX1-MODY, a type of monogenic diabetes known as Maturity Onset Diabetes of the Young (MODY) (3), with symptoms of variable severity including hyperglycemia, pancreatic hypoplasia, general impaired beta cell function, polyhormonal endocrine cells and an increased susceptibility to Type 2 Diabetes (4). The severity of the phenotype and age of onset depend on the specific mutation (5–8). Homozygous *PDX1* mutations in humans lead to pancreatic agenesis and are thus lethal (9).

A well-characterized missense mutation, PDX1^P33T^, located in the transactivation domain, has been studied in human induced pluripotent stem cells (hIPSCs) (5, 10, 11). These *in vitro* studies revealed that the mutation severely impaired the differentiation of progenitors into beta cells, downregulated key target genes, and disrupted beta cell identity, causing transdifferentiation into alpha-like cells. These effects were exacerbated in the homozygous state (10).

While existing mouse models, such as gene knockouts or hemizygous deletions, have advanced our understanding of gene functions (12), they do not recapitulate the specific molecular perturbations of patient point mutations. Such models often overlook critical aspects like residual protein activity, dominant-negative effects or dysregulated-but-present gene expression. They fail to model the genotype–phenotype correlation seen in patients, limiting their translational value. By introducing a defined pathogenic variant into the endogenous locus, disease mechanisms can be studied with precision at the allele level, an essential step toward understanding variant-specific pathology and developing targeted interventions (13).

Given the severe *in vitro* phenotype in hIPSCs and the limitations of current models, we generated a novel mouse model harboring the PDX1^P33T^ mutation. The primary objective of this study was to characterize the *in vivo* consequences of this mutation in the pancreas, glucose homeostasis, and islet function under physiological and metabolic stress conditions.

## 2 Materials and methods

### 2.1 Animals

Mice were kept in a specific-pathogen-free environment in compliance with the Federation of European Laboratory Animal Science Associations (FELASA) protocols and handled according to the recommendations of the Directive 2010/63/EU. Husbandry was in IVC cage systems enriched by mouse houses, bedding material and nestlets. All experiments were performed in accordance with German and European Union guidelines and approved by the government of Upper Bavaria (ROB-55.2-2532.Vet_02-19-152).

Pdx1^P33T^ mice were generated through CRISPR-Cas9 technology and embryo transfer using pure inbred C57BL/6N mouse strain (Charles River) as already described (14). Sequences of gRNA and single-stranded oligonucleotide can be found in **Supplementary Materials**.

Experiments were performed with male and female homozygous *Pdx1* mutant mice denoted as PDX1^P33T^ and wild-type littermates as wild-type. Cohort animals were bred through homozygous intercrosses to reduce animal numbers.

### 2.2 Metabolic studies

Body weight measurements were carried out at *ad libitum* fed state and blood glucose measurements, blood collections, insulin and glucose tolerance tests and final sacrifice were carried out after a period of food deprivation for 6 hours (fasting). HFD (60 kcal% Fat, D12492i, Research Diets Inc.) was fed for 18 weeks, starting at 6 weeks of age.

#### 2.2.1 Glucose and insulin tolerance test

Glucose and insulin tolerance tests were performed at 8 and 20, 12 and 22 weeks of age, respectively. 2 g/kg of glucose (20% solution, 03158931, B.Braun) or 0.75 U/kg human insulin (Huminsulin Normal 100, LILLY Deutschland GmbH) were administered via intraperitoneal (i.p.) injection. Blood glucose values were determined using a blood glucose meter (Contour NEXT ONE) from tail blood at time points 0, 5, 15, 30, 60 and 120 minutes during the glucose tolerance test and time points 0, 15, 30, 60 and 90 minutes during the insulin tolerance test.

#### 2.2.2 Blood collection

At 14 and 24 weeks of age, fasted blood was collected from the retrobulbar plexus in EDTA-containing tubes (16444, Sarstedt).

#### 2.2.3 Body composition analysis

Body composition analysis was done using noninvasive magnetic resonance (MiniSpec LF50, Bruker) to measure lean and fat mass at 18 weeks of age at *ad libitum* fed state.

### 2.3 *In vitro* studies

#### 2.3.1 Islet isolation

Pancreatic islets were isolated as previously describes by (15). Subsequently, they were cultured for 24–48 hours in RPMI medium (21875034, Thermo Fisher) supplemented by 10% v/v fetal bovine serum (26140079, Life Technologies) and 1% v/v penicillin/streptomycin (15140122, Life Technologies) at 37 °C and 5% CO_2_.

#### 2.3.2 ELISA

Insulin content of plasma samples was assessed by Ultra Sensitive Mouse Insulin ELISA (90082, CrystalChem), according to manufacturer’s instructions.

#### 2.3.3 Islet morphology study

Pancreatic tissue was fixed in 4% paraformaldehyde, paraffin-embedded, and sectioned at 3µm. Sections were co-stained for insulin (Cell Signaling #3014; 1:400) and glucagon (Merck G2654; 1:1000) using secondary antibodies AF750 goat anti-rabbit (Invitrogen A21039; 1:100) and AF555 donkey anti-mouse (Invitrogen A32773; 1:200), respectively. Nuclei were counterstained with Hoechst 33342 (Thermo Fisher H1399; 7.5 µg/ml). Sections were scanned at 20x magnification on a Zeiss AxioScan 7 digital slide scanner. Insulin- and glucagon-expressing cell areas were quantified on whole-slide images using Visiopharm image analysis software. beta- and alpha-cell masses were calculated as the percentage of insulin- and glucagon-positive area, respectively, multiplied by the total pancreatic weight. Mean pancreatic islet area was determined from the combined insulin- and glucagon-positive area.

#### 2.3.4 Transcriptome analysis: RNA-sequencing

RNA integrity was verified using a Bioanalyzer 2100 system. Libraries were prepared from mRNA using either non-strand-specific or dUTP-based strand-specific protocols, followed by quality control with Qubit, qPCR, and Bioanalyzer. Pooled libraries were sequenced on an Illumina platform. Raw sequencing reads were quality-filtered with fastp to remove low-quality reads and adapters, then aligned to the reference genome using Hisat2. Gene expression was quantified as FPKM using featureCounts. Differential expression analysis was performed using DESeq2 (for groups with replicates) or edgeR (for groups without replicates). Statistical significance was determined using a Benjamini and Hochberg-adjusted p-value ≤ 0.05 and a log2 fold change ≥ 0.585 (fold change ≥ 1.5). Only genes with a quantitative value in at least 80% of replicates in one condition were included in the analysis.

#### 2.3.5 Proteome analysis

Tissue lysate was subjected to tryptic digestion using the PreOmics iST Kit according to the manufacturer’s protocol. Peptides were analyzed on a Thermo Fisher Scientific VanquishNeo HPLC system coupled to a Bruker timsTOF HT mass spectrometer. Chromatographic separation was performed using an Acclaim PepMap100 C18 trap column and an Aurora C18 analytical column. Peptides were eluted over a 45-minute gradient of 3-40% acetonitrile in 0.1% formic acid. Mass spectrometry data were acquired in DIA-PASEF mode. The acquisition covered a mass range of 300–1250 m/z and a mobility range of 0.65-1.35 1/k_0_. Precursors were isolated with 34 equal windows, applying collision energy from 20 V (0.6 1/k_0_) to 59 V (1.6 1/k_0_). DIA files were processed using Spectronaut (Version 19) as direct DIA. The Pulsar search engine was used against a SwissProt mouse database (Release 2020_02). Quantification was based on MS2 area, using precursor filtering (Qvalue), cross-run normalization, and summing protein-group specific peptide intensities for protein quantification. Only proteins which had a quantitative value in at least 80% of biological replicate, in one condition were included in further analysis. Additionally, only proteins with a *p*-value ≤ 0.05 and log_2_ fold change ≥ 0.585 were considered significantly differentially expressed.

#### 2.3.6 Ingenuity pathway analysis

Significantly altered genes (adj. *p*-value ≤ 0.05) and proteins (*p*-value ≤ 0.05) due to the PDX1^P33T^ mutation were analyzed using Ingenuity Pathway Analysis software (IPA; Qiagen) as previously described (16–21). Each list was analyzed on the basis of predicted molecule classification (other excluded), in addition to canonical pathways and downstream disease and functions, using the IPA *p*-value enrichment score (*p*-value ≤ 0.05) (22).

### 2.4 Statistics and reproducibility

Graphical data were prepared using GraphPad Prism (version 10.0.2 and 10.5.0). Statistical analysis of *in vivo* phenotyping and islet morphology data was performed using one-way or two-way ANOVA followed by the *post hoc* Tukey test. Differentially accumulated genes and proteins were defined as those with a log_2_ fold-change ± 1.5 and adj. *p*-value ≤ 0.05 or *p*-value ≤ 0.05 respectively. Normally distributed data were analyzed by unpaired Student’s *t*-tests to detect differences between wild-type and PDX1^P33T^ islets. Differences between groups were considered significant when *p*-value ≤ 0.05. The number of biological replicates “n” used in each experiment are presented in figure captions, results are presented as mean values ± standard of error of means (SEM) if not indicated otherwise.

## 3 Results

### 3.1 Generation and *in vivo* characterization of a PDX1^P33T^ mouse line

**Figure 1:**
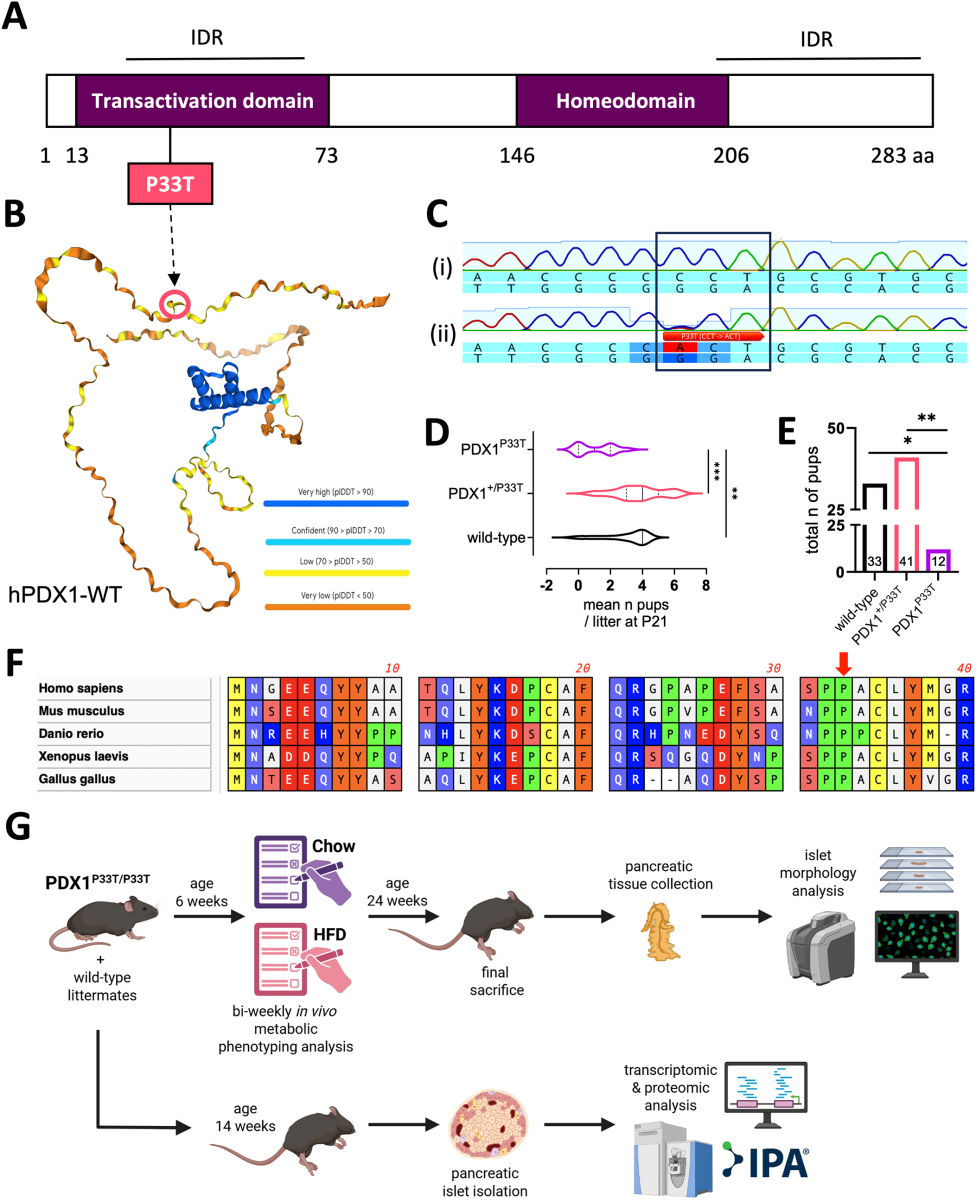
PDX1^P33T^ protein structure does not inhibit viability of homozygous mutant mice. **(A)** Scheme of human PDX1 protein structure displaying its domains, IDRs and the location of the investigated point mutation P33T. **(B)** Alpha-fold rendering of the human PDX1 protein structure, colored according to prediction accuracy certainty, including the location of the investigated point mutation P33T. **(C)** Sequences of the targeted genomic region in the wild-type (i) and founder animal (ii) showing a single nucleotide substitution (C > A) at position c.97. Wild-type sequence: 5’-…CCC**C**CTGCG…-3’. Mutated sequence: 5’-…CCCACTGCG…-3’. **(D)** Graph displaying the mean number of offspring per genotype per litter, at postnatal stage P21, acquired by heterozygous mating (n = 11 litters). **(E)** Graph displaying the total number of pups at postnatal stage P21 acquired from all 11 litters from heterozygous matings, according to their genotypes. Genotype counts deviated significantly from the expected Mendelian 1:2:1 ratio (χ^2^ = 10.44, df = 2, p = 0.0054). When analysis was restricted to surviving offspring (WT vs. Het only), the observed distribution differed from an 1:2 expectation (χ^2^ = 4.22, df = 2, p = 0.04), indicating marked underrepresentation of homozygous and a smaller but detectable reduction in heterozygote survival. **(F)** Multiple sequence alignment of the first 40 amino acids of the PDX1 protein from five different species including homo sapiens (human), mus musculus (mouse), danio rerio (zebrafish), xenopus laevis (frog) and gallus gallus (chicken), particularly depicting the conservation of the P33 residue among multiple species. **(G)** Graphical summary of the experimental pipeline described in this manuscript, HFD – high fat diet, IPA – Ingenuity Pathway Analysis. Created in BioRender (72). "*" p-value ≤ 0.05; "**" p-value ≤ 0.005; "***" p-value ≤ 0.0005.

We generated the PDX1^P33T^ mouse line using CRISPR-Cas9 technology, inducing a C-to-A transversion at position c.97 in exon 1 of the *Pdx1* gene. This causes an amino acid exchange from proline 33 to threonine (P33T) within the N-terminal transactivation domain (TAD), which is located within an intrinsically disordered region (IDR) (**Figures 1A–C**) and is conserved among numerous species (**Figure 1F**). Contrary to our expectations, we observed viable homozygous PDX1^P33T^ offspring following heterozygous intercrosses, though at a significantly decreased Mendelian ratio compared to both wild-type and heterozygous animals (**Figures 1D, E**).

**Figure 2:**
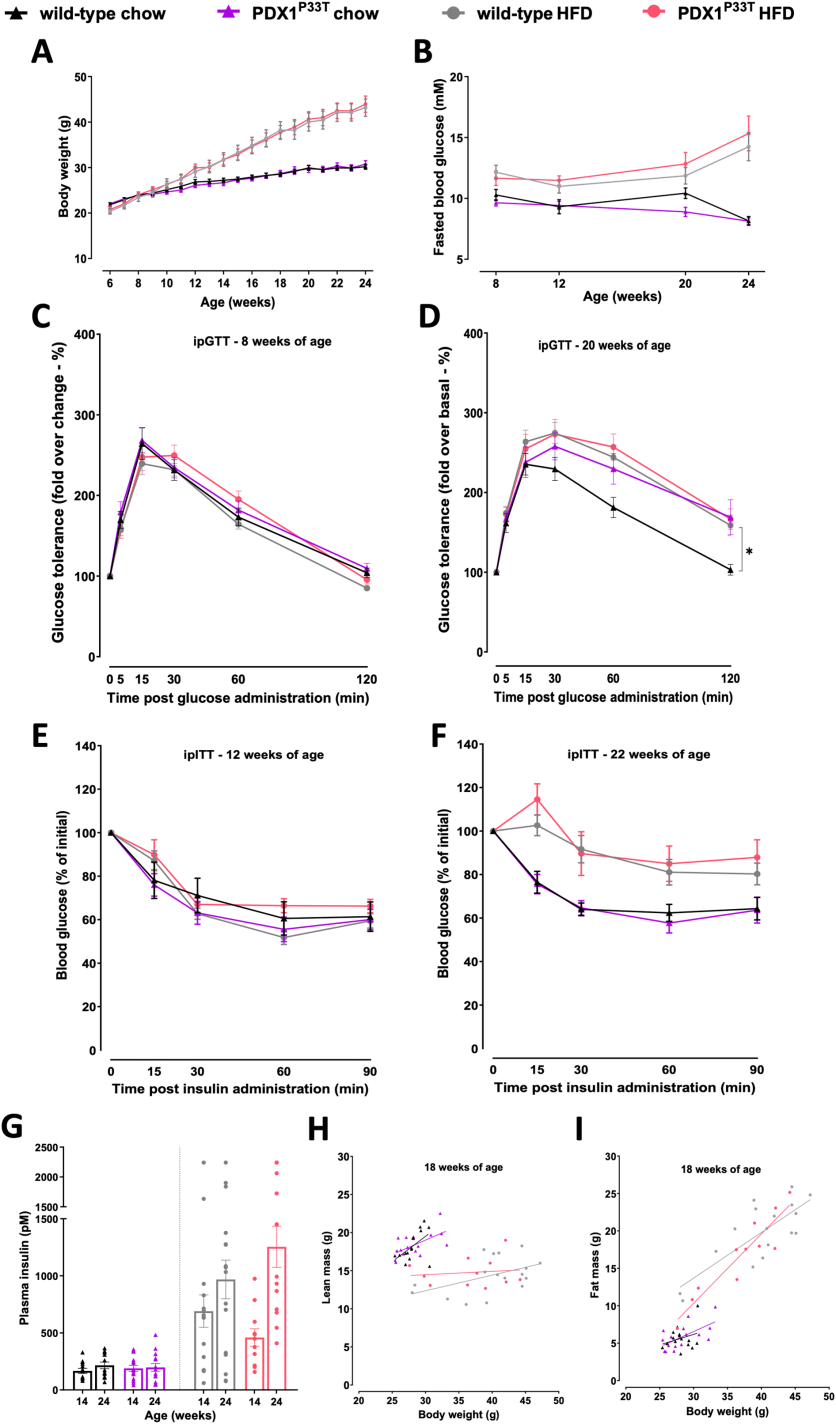
Male PDX1^P33T^ mice maintain normoglycemia and insulin sensitivity in adulthood. Comparison between male wild-type and mutant PDX1^P33T^ mice on either a chow diet or HFD diet. **(A)** Weekly *ad libitum* body weight in wild-type and homozygous PDX1^P33T^ mice, data are shown as mean SEM, n=12-15. **(B)** Blood glucose levels post 6h fasting in wild-type and homozygous PDX1^P33T^ mice, data are shown as mean SEM, n=12-15; **(C)** I.p. glucose tolerance test at 8 weeks of age and **(D)** 20 weeks of age after a 6h fasting period; administered D-glucose (2g/kg); blood glucose values were normalized to baseline (t = 0 min), n=12-15. **(E)** I.p. insulin tolerance test at 12 weeks of age and **(F)** 22 weeks of age after a 6h fasting period; injected with human recombinant insulin (0,75 U/kg); blood glucose values were normalized to baseline (t = 0 min), n=12-15. **(G)** Plasma insulin levels measured in blood samples collected from the retroorbital plexus after a 6h fasting period at 14 and 24 weeks of age from wild-type and PDX1^P33T^ mutant mice, n=12-15. **(H–I)** Body composition analysis showing the linear regression of lean mass **(H)** and fat mass **(I)** against total body weight, n=12-15. "*" p-value ≤ 0.05.

Next, we sought to assess the PDX1-MODY-relevant phenotype and subjected homozygous PDX1^P33T^ mice and their wild-type littermates to a series of phenotyping analyses (**Figure 1G**). To further understand the extent of the phenotype under metabolic stress, we challenged a subset of mice with a high-fat diet (HFD). Routine measurements of body weight (**Figure 2A**) and fasted blood glucose in both male (**Figure 2B**) and female (**Supplementary Figures S1A, B**) mice revealed no changes between the groups, independent of diet and age. To understand whether PDX1^P33T^ mice are responsive to glucose and insulin in adolescence and retain this sensitivity through adulthood we conducted intraperitoneal glucose (i.p.GTT) and insulin tolerance tests (i.p.ITT) at various stages of the phenotyping pipeline. The i.p.GTT at 8 weeks of age (**Figure 2C**, **Supplementary Figure S1C**) remained inconspicuous, though the i.p.GTT at 20 weeks of age in males showed a moderate glucose intolerance on chow diet (**Figure 2D**). Female mice remained glucose tolerant regardless of diet or age (**Supplementary Figure S1D**). Furthermore, the i.p.ITT indicated that at 12 (**Figure 2E**, **Supplementary Figure S1E**) and 22 weeks of age (**Figure 2F**, **Supplementary Figure S1F**) PDX1^P33T^ mice of both sexes remained insulin sensitive.

To assess basal insulin levels as an indicator of metabolic status and possible insulin resistance, we additionally collected fasted blood from the periorbital vein plexus at 14 and 24 weeks of age and measured plasma insulin concentration. At both time points, fasted insulin levels in PDX1^P33T^ mice were comparable to those of wild-type (**Figure 2G**, **Supplementary Figure S1G**). Finally, we investigated the body composition of mutant and wild-type mice on both diets and found no significant differences in fat and lean mass within the parameters of the same diet (**Figures 2H, I**, **Supplementary Figures S1H, I**).

### 3.2 PDX1^P33T^ mice compensate through islet hyperplasia on regular chow diet but fail to adapt on HFD

**Figure 3:**
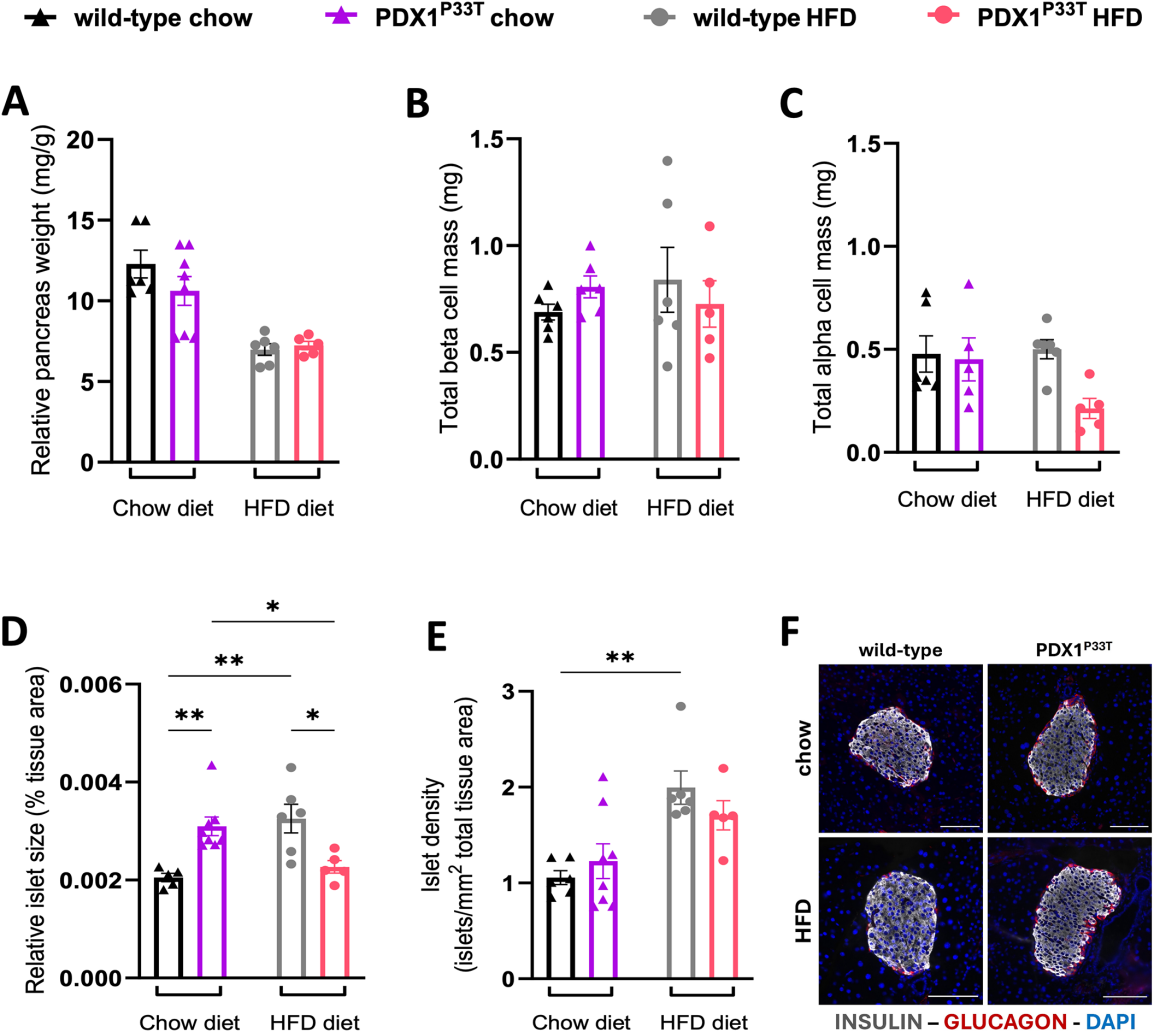
*Pdx1* point mutation does not result in beta cell loss, but rather an increase in islet size. **(A)** Pancreatic tissue weight (mg) of male wild-type and mutant PDX1^P33T^ mice on either a chow diet or HFD diet, normalized to body weight (g), collected at 24 weeks of age. **(B-E)** Bar charts showing the quantification thereof, analyzing total beta cell and alpha cell mass, islet size and islet number on whole pancreatic tissue slices (thickness = 3μm), n=5-8; data are shown as mean with SEM. **(F)** Representative composite images of islets from wild-type and PDX1^P33T^ male mice, from both the chow and HFD cohorts used for the quantification of islet morphology; stained against insulin (gray), glucagon (red) and counterstained with DAPI (blue) to visualize nuclei; images acquired using 20x magnification; scale bar = 100 µm. "*" p-value ≤ 0.05; "**" p-value ≤ 0.005.

To identify whether PDX1^P33T^ mice might be compensating through changes in islet morphology, we used an automated image analysis system to examine islet architecture and composition on pancreatic tissue sections. While there were no relative differences in pancreas weight and beta cell and alpha cell hypertrophy between PDX1^P33T^ mice and wild-type (**Figures 3A–C**, **Supplementary Figures S2A–C**), we observed that male mutant mice under chow diet conditions had larger islets than the control group (**Figure 3D**). However, under HFD conditions, male PDX1^P33T^ mice fail to increase islet size in response to the metabolic challenge to the same extent observed in wild-type (**Figure 3D**). Furthermore, the control group showed a diet-dependent increase in islet number, while mutant mice did not (**Figure 3E**). Both changes cannot be observed in female PDX1^P33T^ mice (**Supplementary Figures S2D, E**). Additionally, we observed no significant changes in islet architecture with no shift in relative prevalence in beta- and alpha-cells determined by insulin and glucagon expression, respectively, or the presence of double positive for both hormones cells (**Figure 3F**, **Supplementary Figures S2F**, **S3A, B**).

### 3.3 Subtle molecular shifts in PDX1^P33T^ islets hint at increased diabetes susceptibility

To uncover the molecular basis for the absence of a diabetic phenotype in male PDX1^P33T^ mice but existing changes in islet morphology, we conducted concurrent bulk RNA-sequencing and proteomics analyses on whole pancreatic islets isolated from chow-fed 14-week-old male PDX1^P33T^ and wild-type mice. This time point, equivalent to approximately 30 human years (23), sufficiently covers the clinical onset period in human patients (24).

**Figure 4:**
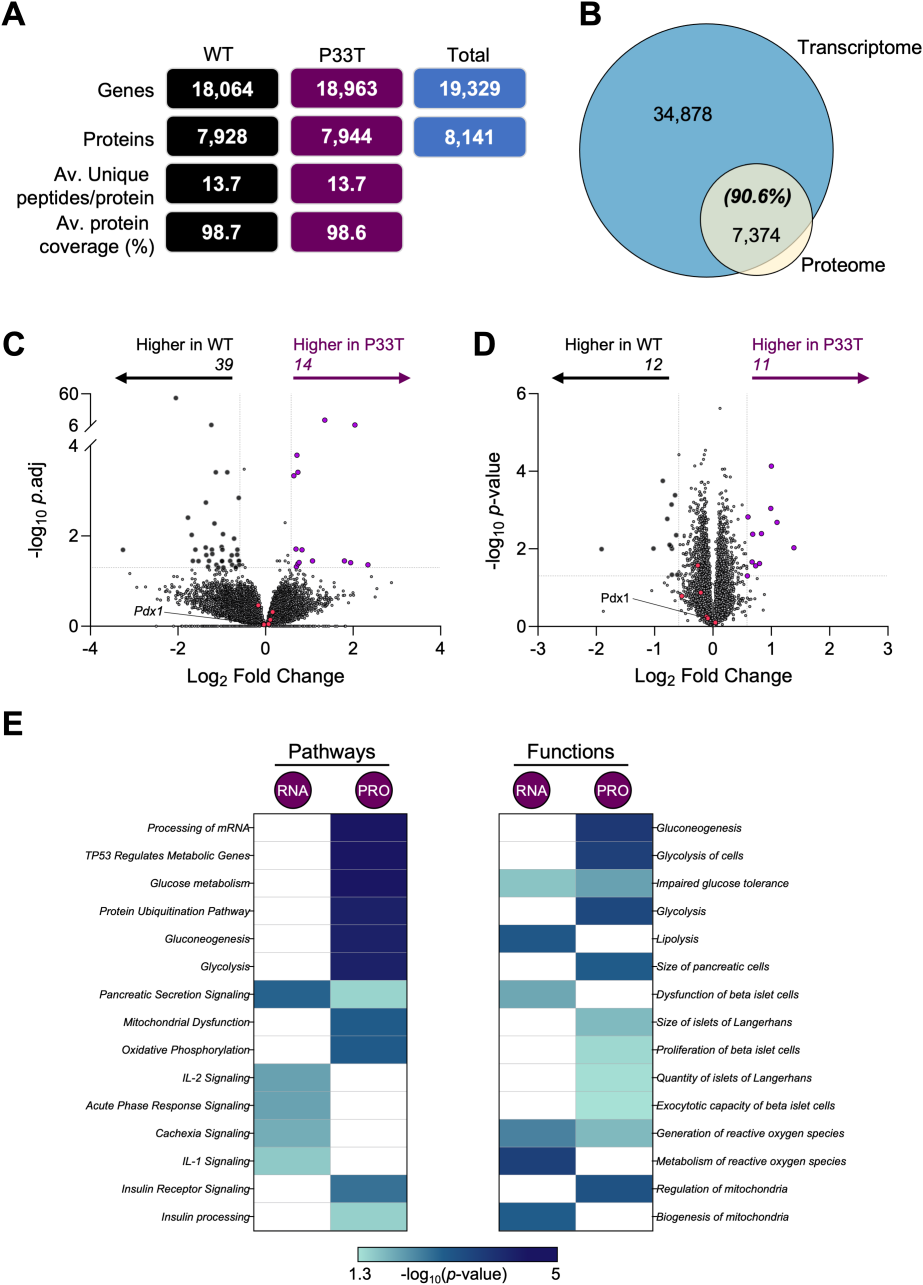
Transcriptomic and proteomic profiles of whole pancreatic islets of PDX1^P33T^ mice show hints at increased cell stress. Transcriptomic and proteomic analyses were performed using isolated islets as described above (n = 5–6 biological replicates collected from 6 individual mice/biological replicate). **(A)** The total number of genes and proteins identified within each population and total, including the average number of unique peptides per protein and average protein coverage. **(B)** Overlap between all gene-protein products and those detected in the proteomic analysis. Volcano plots depict the log_2_ fold change (x-axis) in **(C)** gene and **(D)** protein expression; y-axis is the –log_10_ adj. or *p*-value respectively. Genes/proteins with significantly different expression (fold change ± 1.5 and *p*-value ≤ 0.05) are drawn in black (downregulated) and purple (upregulated). Genes/proteins of interest (Pdx1, Kcnj11, Mafa, Slc2a2, Ins1, Ins2) are colored in pink. **(E)** Heatmaps present the top 15 significantly enriched (*p*-value ≤ 0.05) pathways and functions identified by Ingenuity Pathway Analysis.

Initial RNA-sequencing returned a complex core of 19,329 genes, with 18,064 in wild-type males and 18,963 in PDX1^P33T^ males (**Figure 4A**, **Supplementary Table S1**). Protein-coding genes comprised the largest fraction (79.7%), followed by long non-coding RNAs (11.8%), and processed pseudogenes (5.4%) (**Supplementary Figure S4A**). A direct comparison found that a substantial 1,265 genes were uniquely detected in PDX1^P33T^ mice, while a comparative smaller 366 genes were absence (i.e. wild-type only) (**Supplementary Figure S4B**). Additionally, data independent acquisition (DIA) proteomics was performed, revealing a suite of proteins, totally 8,141 (**Figure 4A**, **Supplementary Table S2**). This was accompanied by high protein coverage, averaging 98.7% across all mice, with 13.7 unique peptides per protein. A direct comparison of the protein identifications revealed a core proteomic inventory of 7,731, with 213 proteins uniquely detected in PDX1^P33T^ mice and 197 protein absence (**Supplementary Figure S4C**).

Protein identifications were integrated with UniProt for annotation (**Supplementary Figure S4D**). Moreover, we found >90% of the proteome was captured in the transcriptome data (**Figure 4B**, **Supplementary Tables S1, S2**). Initial interrogation of each dataset with Ingenuity Pathway Analysis (IPA) demonstrated no overt differences in the categorizations of molecule type, except the RNA level invested more in transcription regulators (19.2 v 15.5%) and receptors (2.6 v 6.6%), while the protein level invested in enzymes (56.3 v 64.7%) (**Supplementary Figure S4E**). Only three proteins were significantly altered (FC ± 1.5, *p*-value ≤ 0.05) in both datasets, oxysterol-binding protein-related protein 5 (Osbpl5), jupiter microtubule associated homolog 1 (Jpt1) and FXYD domain-containing ion transport regulator 6 (Fxyd6).

To delve deeper into the quantitative expression differences within each omics, we compared the profiles from PDX1^P33T^ and wild-type islets (**Supplementary Tables S1, 2**). Volcano plot analysis revealed no significant dysregulation at the RNA (FC ± 1.5, adj *p*-value ≤ 0.05) or protein level (FC ± 1.5, *p*-value ≤ 0.05) of *Pdx1* itself or known PDX1 targets such as insulin 1(*Ins1*), MAF bZIP transcription factor A (*Mafa*) and solute carrier family 2 (facilitated glucose transporter), member 2 (*Slc2a2*) (**Figures 4C, D**, **Supplementary Tables S1, S2**). We did observe a very modest shift in gene expression with 14 significantly upregulated, including T cell specific GTPase 1 (*Tgtp1*), cGMP-dependent protein kinase 2 (*Prkg2*) and immunoglobulin mu Fc receptor (*Fcmr*) (**Figure 4C**, **Supplementary Table S1**). Conversely, 39 genes were significantly downregulated, including X-linked lymphocyte regulated gene 4 (*Xlr4b*), *Osbpl5*, and tumor necrosis factor receptor superfamily member 16 (*Ngfr*). In accordance with the RNA analysis, the proteomic volcano plot similarly indicated modest changes, with only 23 proteins significantly dysregulated (**Figure 4D**, **Supplementary Table S2**). The largest changes observed were associated with C->U-editing enzyme APOBEC-2, protein unc-13 homolog A (Unc13A) and small integral membrane protein 15 (Smim15).

To contextualize these subtle changes, we employed IPA to investigate associated pathways and downstream molecular functions (**Figure 4E**, **Supplementary Table S3**). Three overarching categories were identified: pancreatic and hormonal signaling, core metabolic circuitry, and mitochondrial/oxidative processes. For pathways, the most striking shared hit was *Pancreatic Secretion Signaling*. At the protein level, downstream insulin action emerged through *Insulin Receptor Signaling* and at the RNA level, *PPAR Signaling*, a master regulator of lipid and glucose homeostasis, featured prominently. *Glucose Metabolism* and its anaplerotic branch, the *Pentose Phosphate Pathway*, were significantly enriched, with the latter predicted to be inhibited (Z score = -2.65), suggesting a proteomic down-regulation of key glycolytic and NADPH-generating enzymes. A broader, tumor-suppressor-linked node, *TP53 Regulates Metabolic Genes*, surfaced as well. Lastly, mitochondrial bioenergetics were captured by *Respiratory Electron Transport* together with *Oxidative Phosphorylation*, both predicted to be activated (Z score = 3.60). Consistently, IPA also predicted inhibition of *Mitochondrial Dysfunction* (**Figure 4E**, **Supplementary Table S3**).

For molecular functions and disease, the most prominent pancreatic hits were *Endoplasmic-Reticulum in Pancreatic Cells*, *Size & Quantity of islets, Insulin Resistance*, and *Impaired Glucose Tolerance* (**Figure 4E**, **Supplementary Table S3**). Consistent with the metabolic pathway cluster, the top hits were *Glucose Metabolism Disorder*, *Biosynthesis of Glyceraldehyde-3-phosphate*, *Gluconeogenesis*, and *Glycolysis*. Lastly, for the mitochondrial category, we observed *Synthesis and Metabolism of ROS*, accompanied by *Mitochondria Biogenesis*.

## 4 Discussion

This study reports the characterization of a novel mouse model for PDX1-MODY. Unlike previously described murine models (25–31), this model closely mimics the human genetic cause as it carries a well-characterized pathogenic point mutation found in human patients, P33T (10). Surprisingly, our primary findings indicate an unexpected discrepancy in the phenotypic expression of this MODY mutation in mice, causing them to diverge from human pathology and represent a striking exception to the current literature.

As an essential transcription factor for beta cells (32), PDX1 regulates the expression of several key genes including *Ins1*, *Ins2*, *Mafa*, *Neurod1* and even itself. Moreover, PDX1 acts as a master regulator of pancreatic development, ensuring pancreatic progenitor cell commitment to a beta cell identity (30, 32). The complete loss of *Pdx1* in mice, achieved through homozygous knockout models, leads to pancreatic agenesis and is lethal a few days after birth (9, 33, 34). Previously described heterozygous *Pdx1* knockouts often show glucose intolerance and reduced insulin secretion in response to glucose, partially mimicking the phenotype in human PDX1-MODY patients (25, 27). The functional activity of PDX1 is largely controlled by its intrinsically disordered N-terminal and C-terminal regions, which give it the required flexibility for binding a great number of different co-factors (**Figure 1B**). The N-terminal transactivation domain (residues 13–73) enables interactions with key co-factors, including p300/CBP, Set7 methyltransferase, Bridge-1, and the transcription factor complex E47/beta2. It also supports recruitment of the Brg1-containing Swi/Snf chromatin remodeling complex (35). These interactions are strongly influenced by glucose-dependent post-translational modifications, in particular phosphorylation and methylation, which emphasize the central regulatory role of the disordered regions in PDX1-mediated transcription (35).

The P33T mutation in the transactivation domain of PDX1 replaces a structurally rigid proline with a polar threonine, which may introduce significant changes in local structure and flexibility (36). This substitution can affect how PDX1 interacts with cofactors, particularly p300 and other chromatin-modifying enzymes, and may interfere with normal phosphorylation patterns in the domain. As a result, the mutation has the potential to weaken the transcriptional activity of PDX1, which could contribute to the development of MODY (10). Complete deletions of PDX1 are known to cause post-natal lethality, however, contrary to our expectations, the survival of homozygous PDX1^P33T^offspring was not tremendously affected, though the Mendelian ratio of pups born from PDX1^+/P33T^ het x het intercrosses was skewed with a significantly smaller number of homozygous offspring (**Figures 1D, E**). This significant deviation from the expected Mendelian distribution (37, 38) is a critical finding in itself. It strongly suggests that the P33T mutation, when homozygous, could produce a developmental defect that leads to embryonic lethality in a substantial portion of the animals, and confers a milder but biologically relevant reduction in heterozygote survival, potentially reflecting haploinsufficiency or a dominant-negative effect of the mutant protein. Given the indispensable role of PDX1 in pancreatic organogenesis, such an outcome is highly plausible. While a detailed investigation into the mechanisms of this developmental phenotype is beyond the scope of our present study, which focuses on the postnatal metabolic consequences in surviving animals, elucidating the precise timing and cellular basis of this partial lethality represents a critical and exciting avenue for future investigation.

Although all known human PDX1^P33T^ MODY mutations are heterozygous (32), hIPSCs carrying a homozygous PDX1^P33T^ mutation remain vital but with a stronger phenotypic impact (10). Therefore, we decided to proceed with a cohort of homozygous PDX1^P33T^ mice aiming to investigate the expectedly more severe phenotype. Additionally, a secondary cohort was challenged by a HFD to potentially exacerbate the diabetic phenotype through metabolic stress (39).

The non-ketogenic nature of MODY is reflected by the lack of altered body weight in the PDX1^P33T^ mice, however, the lack of hyperglycemia was unanticipated based on existing knowledge (6, 7, 40). Furthermore, the modestly reduced glucose tolerance in male PDX1^P33T^ mice on chow at 20 weeks of age but normal insulin levels in PDX1^P33T^ mice independent of diet, sex, and age are results that deviate markedly from prior expectations, since PDX1 is crucial for both processes. It binds the insulin gene promoter, facilitating its transcription in response to varying glucose levels (41) and loss or mutations of PDX1 are known to impair this process causing glucose intolerance (32, 42). Moreover, PDX1 controls key elements involved in glucose uptake and metabolism (like GLUT2 and GCK), thereby enabling the subsequent triggering of insulin secretion in response (43). In our mice, we expected impairment of both mechanisms. PDX1 also supports the adaptive expansion of beta cell mass under metabolic stress, including HFD (31, 44). Morphological analysis revealed a significant increase in relative islet size in male PDX1^P33T^ mice compared to wild-type under standard chow diet conditions. This expansion occurs despite the maintenance of preserved glucose homeostasis and the absence of overt insulin resistance, demonstrating a notable compensatory response in the PDX1^P33T^ mouse pancreas. Unlike hyperplasia driven by systemic insulin resistance (45–47), this finding, paired with the modest glucose intolerance, suggests an early adaptive remodeling phase in response to the PDX1 point mutation. The robust compensatory capacity of the murine pancreas strongly supports such an adaptive mechanism (48, 49). Interestingly, a comparable morphological adaptation could not be determined in female PDX1^P33T^ mice. This attenuated phenotype and supposed protection in female mice is likely due to the combined beneficial effects of estrogen on β-cell health and systemic insulin sensitivity, as well as an inherent pancreatic plasticity evolved to cope with the metabolic demands of pregnancy (50–54).

To explore the molecular basis of this adaptation observed in male PDX1^P33T^ mice, we performed bulk RNA-sequencing and complementary proteomic analysis on isolated islets. Despite the P33T mutation, neither PDX1 itself nor its canonical targets (*Ins1*, *Mafa*, *Slc2a2*) were dysregulated, indicating that PDX1 protein stability was not compromised by the mutation (55, 56). This finding rather suggests the P33T substitution may only partially impair transcriptional co-factor binding without critically disrupting PDX1’s DNA-binding affinity or nuclear localization, as observed in prior models (9, 57). Preservation of these essential gene expressions fits with the sustained insulin secretion and glucose homeostasis.

The identification of 1,265 unique transcripts and 213 proteins exclusively in PDX1^P33T^ mice (**Supplementary Figures S4C, D**) as well 14 upregulated and 39 downregulated genes with significant expression shifts, highlights an islet environment undergoing adaptive remodeling. Notably, these genes and proteins are not overtly linked to beta cell failure, but rather cluster into immune modulation (*Tgtp1*, *Fcmr*), ion transport (*Fxyd6*), and mitochondrial regulation (*Unc13A*, *Osbpl5*), suggesting reprogramming toward stress resilience. These molecular observations are consistent with the normoglycemic phenotype in mice, but stands in contrast to observations previously made in hIPSCs carrying the identical PDX1^P33T^ mutation, where analysis revealed widespread downregulation of beta cell specific genes and upregulation of alpha cell associated genes, indicating a tendency toward transdifferentiation from insulin- to glucagon-producing cells (10). This discrepancy emphasizes the substantial differences between these model systems and highlights the limitations of directly translating findings from simplified *in vitro* models to the pancreatic environment complex *in vivo*, particularly between species.

Pathway enrichment via IPA further reveals an adaptive metabolic shift rather than a dysfunction signal. A key feature of the molecular analysis is the concerted upregulation of mitochondrial respiratory function with inhibition of mitochondrial dysfunction, activation of oxidative phosphorylation and the electron transport chain pathways (Z score = +3.60), implying a hyperfunctional state to boost ATP synthesis. These factors are critical for long-term viability of beta cells (58, 59).

Enrichment of Glucose Metabolism, Glycolysis, and Gluconeogenesis pathways indicates a broad reconfiguration of intermediary metabolism. This includes suppression of the Pentose Phosphate Pathway (PPP) (Z score = –2.65). Despite normal fasting glucose *in vivo*, functional annotations predict activation of ER stress, TP53-regulated metabolic reprogramming (60), and impaired glucose tolerance. These *in silico* predictions, although not phenotypically validated, suggest a pre-emptive state of the islet in which adaptive stress responses are engaged to maintain homeostasis. Finally, the noteworthy enrichment of pathways like Proliferation of β-cells, Quantity of islets of Langerhans and Size of islets of Langerhans, further support the morphological phenotype of islet hyperplasia seen in these mice independently, on a molecular level.

Notably, the expansion in islet size and number is absent under HFD, suggesting impaired adaptation to metabolic stress. The mutants show normal islet numbers and even a reduced average size, indicating diet-dependent suppression or normalization of islet remodeling and likely reflecting an override of genetically induced islet adaptations by environmental influences. Combined, the morphological evidence of basal compensation on chow and the impaired adaptive growth on HFD, supported by the molecular signatures revealed by omics analysis, indicate a model wherein early compensatory remodeling in PDX1^P33T^ islets preserves endocrine function under baseline conditions but fails to scale or persist under chronic dietary stress, which ultimately homogenizes metabolic outcomes across genotypes.

PDX1 is a key transcription factor required for pancreatic development in both mice and humans and analysis of PDX1 orthologs revealed a very high degree of protein conservation, and notably, the absolute conservation of the proline at position 33 (P33), highlighting the likely functional constraints on this residue (Uniprot (61) ID 52946 (human PDX1), ID 52946 (mouse PDX1) (**Figure 1F**). However, important species-specific differences in its regulation and chromatin context have been identified that likely contribute to the divergent phenotypes observed between model systems. In mice, PDX1 is indispensable for early pancreatic bud formation, acting through a well-characterized enhancer landscape that becomes active during foregut patterning (62). In contrast, in humans, PDX1 operates within a distinct regulatory framework involving primed, lineage-specific enhancers and chromatin looping interactions that do not appear to be conserved in the mouse (63). Only ~8% of PDX1 binding sites are conserved between species, yet approximately 84% of the genes bound by PDX1 in human islets are also bound in mouse islets, indicating a conserved target gene repertoire but divergent enhancer selection (64). Comparison of PDX1 binding sites between human and mouse revealed both conserved and species-specific binding events, indicating evolutionary conservation of core regulatory networks (like the *Insulin* gene (65)) as well as divergence in enhancer usage (66). Notably, human PDX1 shows a preference for binding intronic and intergenic regions, whereas murine PDX1 more frequently binds promoter-proximal sites, highlighting species-specific differences in the use of cis-regulatory elements and transcriptional control logic (64). These interspecies distinctions in enhancer architecture and transcriptional targeting likely contribute to the differences observed when modeling human beta cell development in murine systems (32, 63). However, this widespread divergence in the downstream target enhancers of PDX1 contrasts sharply with the evolutionary conservation of the regulatory regions that control the expression of the *pdx1* gene itself. Early *in vivo* analysis by Gannon et al. identified a localized region upstream of the mouse *pdx1* gene that is remarkably conserved in sequence between vertebrates (67). Compensatory mechanisms such as beta cell neogenesis from ductal precursors and adaptive endoplasmic reticulum stress responses have been shown to preserve beta cell function and mass in mouse models of diet-induced diabetes (13). Furthermore, extreme beta cell loss can trigger alpha-to-beta cell transdifferentiation, underscoring the plasticity of islet cells in mice (68). We did not observe either processes in our analysis.

In the past, numerous mouse models carrying mutations in genes responsible for MODY have been generated, providing useful comparisons for our study. For example, heterozygous *Hnf1a* knockout mice display only mild glucose intolerance and do not fully reproduce the diabetes phenotype seen in humans with HNF1A-MODY (69). Similarly, Hnf4a mutant mice show variable defects in insulin secretion and lipid metabolism, again with incomplete overlap with the human phenotype (70, 71). Mouse models of Pdx1 mutations also highlight species differences: while homozygous loss leads to pancreatic agenesis, heterozygous mice develop only mild glucose intolerance rather than overt diabetes, in contrast to the progressive β-cell failure characteristic of PDX1-MODY in humans (9, 25, 33). Together, these examples illustrate that while MODY genes are highly conserved, mouse models frequently show milder or divergent phenotypes compared to patients. This discrepancy underscores important species-specific differences in β-cell physiology and gene regulatory networks, which may explain why our particular model did not recapitulate the expected human phenotype.

While this study characterized the islet phenotype of PDX1^P33T^ mice and provided initial molecular insights into the respective transcriptomic and proteomic profiles, further investigations are warranted to fully elucidate the mechanisms underlying the observed compensation and impaired adaptive capacity. Given PDX1’s role as a transcription factor and the P33T mutation in a functional domain, it is crucial to understand how this specifically affects its molecular activity. Further studies employing techniques like chromatin immunoprecipitation sequencing (ChIP-Seq) could determine whether the mutation alters the DNA binding profile of PDX1, potentially explaining subtle transcriptional changes. Co-immunoprecipitation mass spectrometry could reveal effects on protein interactions and transcriptional complex formation. Finally, elucidating the long-term consequences of the impaired adaptive response observed on a high-fat diet would require extending the observation period in future studies to determine whether this leads to a decompensated state and overt diabetes pathology over time.

In summary, our characterization of a mouse model with the human MODY-causing PDX1^P33T^ mutation revealed a striking divergence from the human phenotype and highlighted the importance of PDX1’s disordered regions in mediating dynamic protein-protein interactions essential for insulin expression. Future mechanistic investigations are crucial to understand the precise molecular basis for this divergence and its implications for human health.

## Data Availability

The mass spectrometry proteomics data have been deposited to the ProteomeXchange Consortium (http://proteomecentral.proteomexchange.org) via the PRIDE partner repository with the dataset identifier PXD066252. The RNA-sequencing data have been deposited to the Gene Expression Omnibus (GEO) data repository with the dataset accession GSE303047.
