# Supplementary material for: MODY PDX1P33T: a mouse model reveals phenotypic divergence from human disease
Source: Front Endocrinol (Lausanne). 2025 Oct 22;16:1680893. doi: 10.3389/fendo.2025.1680893 (PMC12585948; doi:10.3389/fendo.2025.1680893)
Supplement: Supplementary file 1 [file DataSheet1.docx]

Supplementary Material

# Supplementary Data

# The Pdx1^P33T^ mouse line was generated using CRISPR-Cas9-based gene editing of one-cell embryos. Before electroporation, a specific single guide RNA (Pdx1_P33T_gRNA: 5′-CGGCCCATGTACAGGCACGC-3′, Metabion), the sgRNA (200 ng/µl), and single-strand oligonucleotides (ssODN_Pdx1_P33T_E814D: 5´-CTCTACAAGGACCCGTGCGCATTCCAGAGGGGCCCGGTGCCAGAGTTCAGCGCTAACCCCACTGCGTGCCTGTACATGGGCCGCCAGCCCCCACCTCCGCCGCCACCCCAGTTTACAAGC-3´; 300 ng/μl) were diluted in Opti-MEM buffer (Thermo Fisher Scientific) along with recombinant Cas9 protein (200 ng/μl, IDT). The mixture was incubated for 10 minutes at 20 °C, followed by 10 minutes at 37 °C, to form the active ribonucleoprotein complex. One-cell embryos were obtained by mating C57BL/6N males (Charles River) with C57BL/6N females, which were super-ovulated with 5 U of pregnant mare’s serum gonadotropin and 5 U of human chorionic gonadotropin. The embryos were then electroporated using an NEPA21 electroporator with a CUY501P1-1.5 electrode (Nepa Gene Co). The resulting zygotes were transferred into pseudopregnant CD1 female mice to obtain live pups. Gene-editing events were analyzed using genomic DNA isolated from ear biopsies of founder mice and F1 progeny. DNA extraction was performed using the Wizard Genomic DNA Purification Kit (Promega, A1120), following the manufacturer’s instructions.

# Supplementary Figures and Tables

## Supplementary Figures

**Supplementary Figure 1: Female PDX1^P33T^ mice maintain normoglycemia and insulin sensitivity in adulthood.** Comparison between female wild-type and mutant PDX1^P33T^ mice on either a chow diet or HFD diet. **(A)** Weekly *ad libitum* body weight in wild-type and homozygous PDX1^P33T^ mice, data are shown as mean SEM, n=12-15. **(B)** Blood glucose levels post 6h fasting in wild-type and homozygous PDX1^P33T^ mice, data are shown as mean SEM, n=12-15; **(C)** I.p. glucose tolerance test at 8 weeks of age and **(D)** 20 weeks of age after a 6h fasting period; administered D-glucose (2g/kg); blood glucose values were normalized to baseline (t = 0 min), n=12-15. **(E)** I.p. insulin tolerance test at 12 weeks of age and **(F)** 22 weeks of age after a 6h fasting period; injected with human recombinant insulin (0,75 U/kg); blood glucose values were normalized to baseline (t = 0 min), n=12-15. **(G)** Plasma insulin levels measured in blood samples collected from the retroorbital plexus after a 6h fasting period at 14 and 24 weeks of age from wild-type and PDX1^P33T^ mutant mice, n=12-15. **(H-I)** Body composition analysis showing the linear regression of lean mass (H) and fat mass (I) against total body weight, n=12-15.

**Supplementary Figure 2: *Pdx1* point mutation does not affect islet morphology in female mice**. **(A)** Pancreatic tissue weight (mg) of female wild-type and mutant PDX1^P33T^ mice on either a chow diet or HFD diet, normalized to body weight (g), collected at 24 weeks of age. **(B-E)** Bar charts showing the quantification thereof, analyzing total beta cell and alpha cell mass, islet size and islet number on whole pancreatic tissue slices (thickness = 3μm), n=5-7; data are shown as mean with SEM. **(F)** Representative composite images of islets from wild-type and PDX1^P33T^ female mice, from both the chow and HFD cohorts used for the quantification of islet morphology; stained against insulin (grey), glucagon (red) and counterstained with DAPI (blue) to visualize nuclei; images acquired using 20x magnification; scale bar = 100 µm.

**Supplementary Figure 3: Representative images of the unchanged islet morphology of wild-type and PDX1^P33T^ male and female mice.** Representative immunofluorescent images of pancreatic islets from **(A)** male and **(B)** female wild-type and PDX1^P33T^ mice from both the chow and HFD cohort used for the quantification of islet morphology; stained against insulin (grey), glucagon (red) and counterstained with DAPI (blue) to visualize the nuclei; image acquired using 20x magnification; scale bar = 100 µm.

**Supplementary Figure 4: Transcriptomic and proteomic profiles of whole pancreatic islets of male PDX1^P33T^ mice.** Transcriptomic and proteomic analyses were performed using isolated islets as described above (n = 5-6 biological replicates collected from 6 individual mice/biological replicate). **(A)** A pie chart showing the annotation of gene biotypes discovered in the initial RNA-sequencing core dataset. **(B-C)** Venn diagrams showing a direct comparison of common and unique to the wild-type and PDX1^P33T^ mutant datasets genes (B) and proteins (C), respectively. **(D)** Stacked bar chart showing the percentage of identified proteins based on their evidence level for existence (protein, transcript and none, thus inferred from homology) within the UniProt knowledgebase. **(E)** Stacked bar chart showing the functional categorization of molecules identified at bothe the RNA and protein levels, as analyzed by IPA. The proportions for each category are represented as a percentage of the total number of molecules in each dataset.

## Supplementary Tables

**Table S1**

Refined transcriptomic list of all genes detected in male wildtype and PDX1^P33T^ beta islets, as well, as a quantitative comparison.

**Table S2**

Refined proteomic list of all protein detected in male wildtype and PDX1^P33T^ beta islets, as well, as a quantitative comparison.

**Table S3**

Ingenuity Pathway Analysis outputs for significantly altered genes and proteins in male PDX1^P33T^ beta islets.
