# Supplementary figures and images for: MODY PDX1P33T: a mouse model reveals phenotypic divergence from human disease
Source: Front Endocrinol (Lausanne). 2025 Oct 22;16:1680893. doi: 10.3389/fendo.2025.1680893 (PMC12585948; doi:10.3389/fendo.2025.1680893)

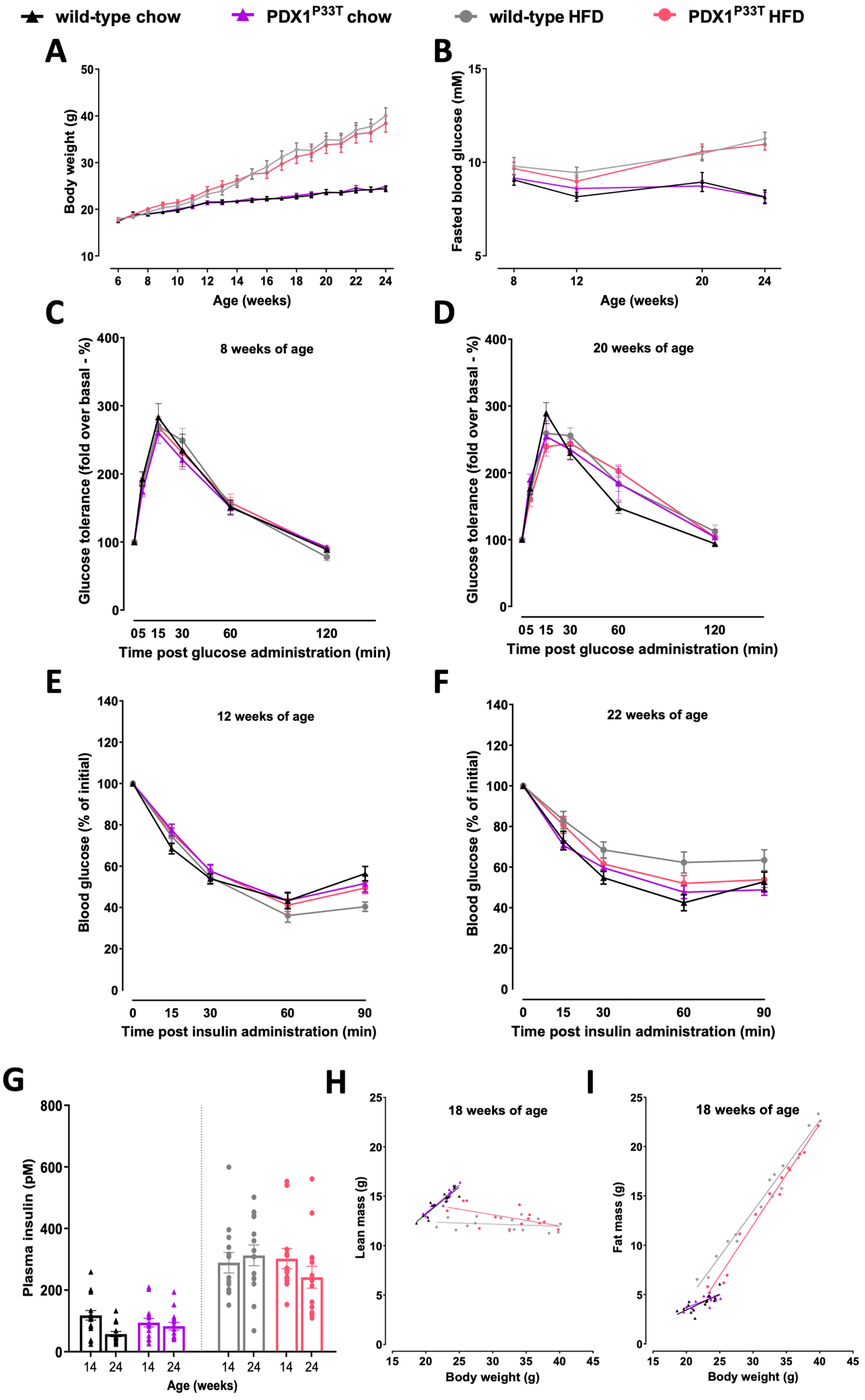

Supplement: Supplementary file 2 [file Image1.tif]

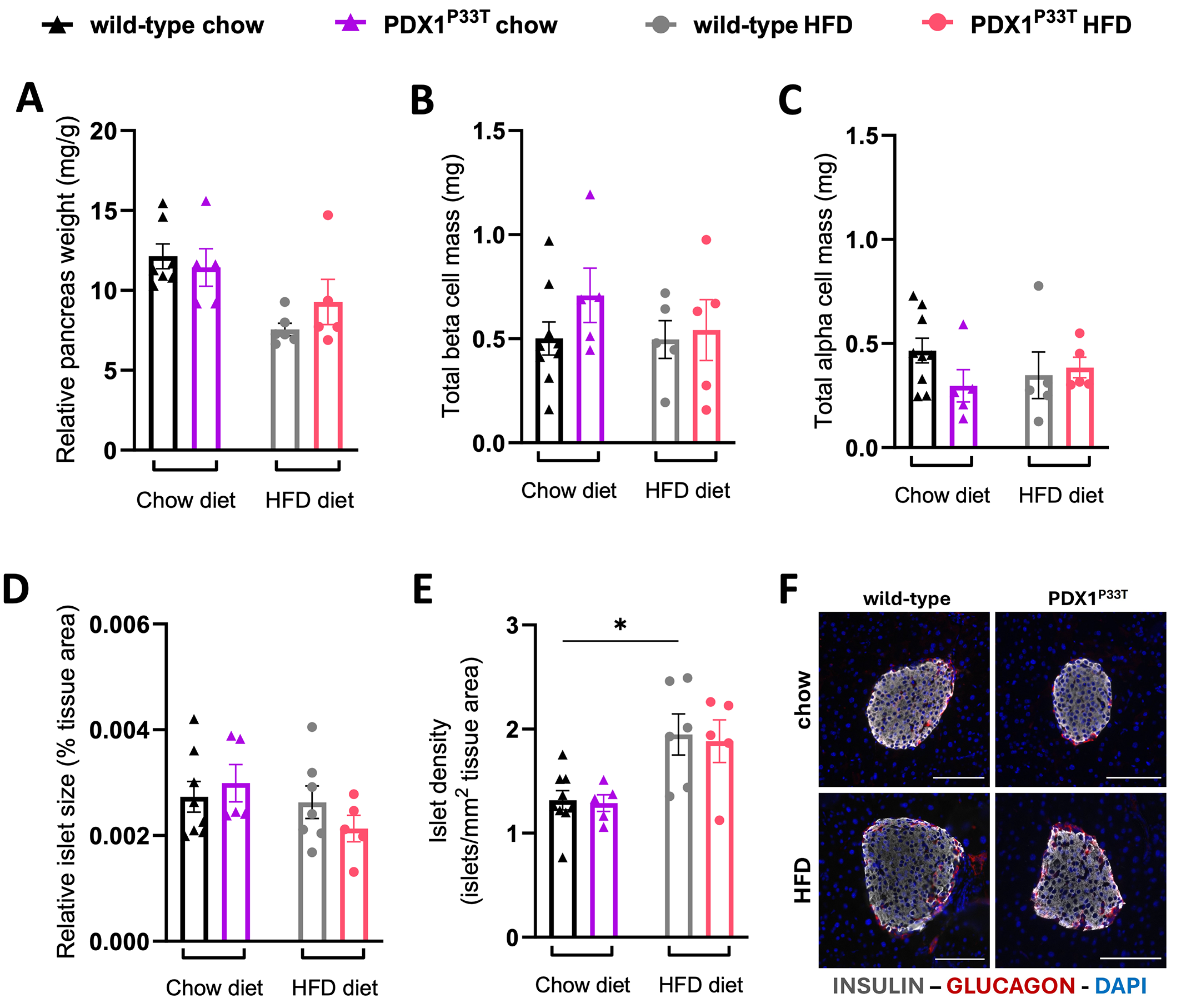

Supplement: Supplementary file 3 [file Image2.tif]

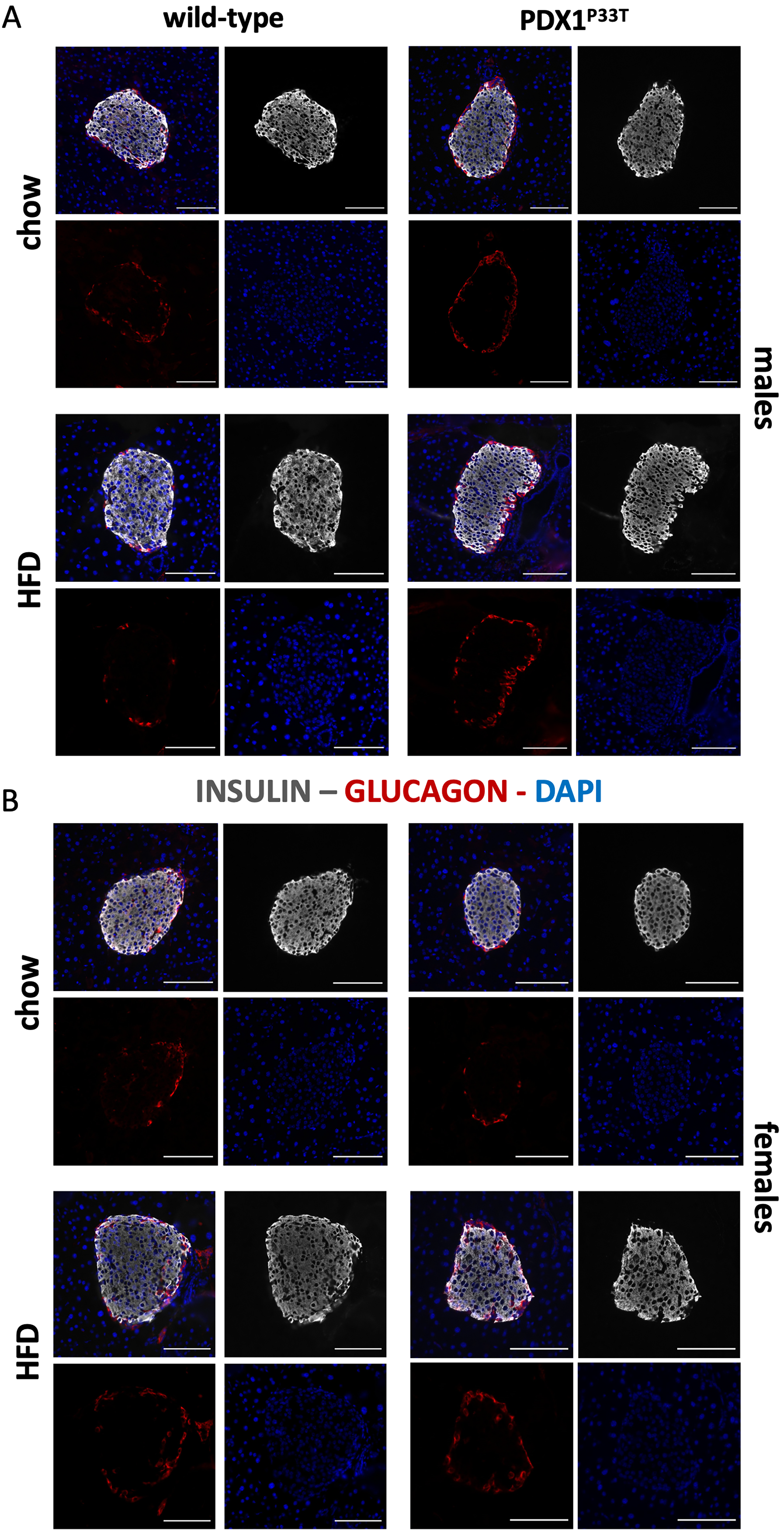

Supplement: Supplementary file 4 [file Image3.tif]

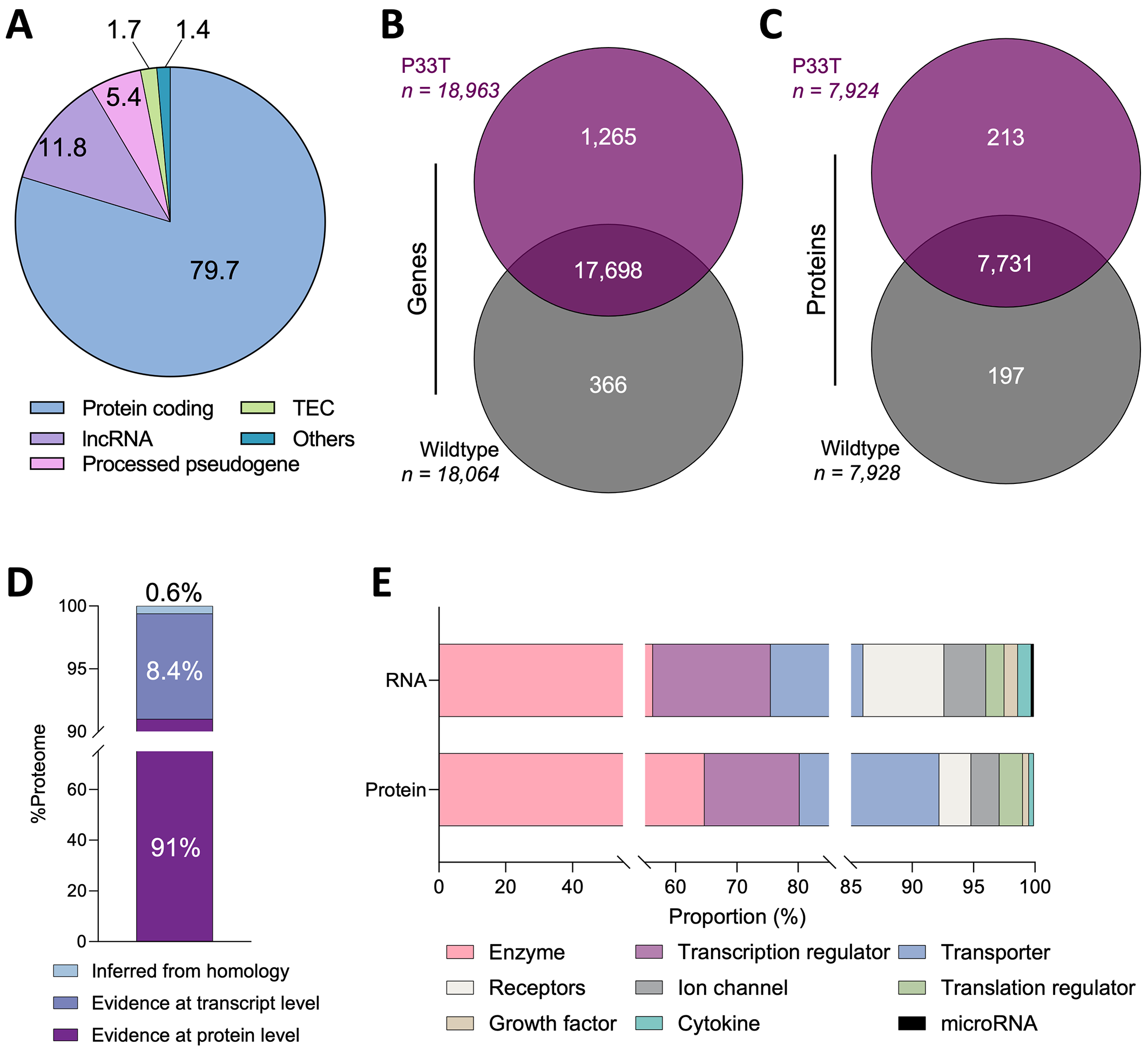

Supplement: Supplementary file 5 [file Image4.tif]
